# Implementing Public Health Strategies—The Need for Educational Initiatives: A Systematic Review

**DOI:** 10.3390/ijerph18115888

**Published:** 2021-05-30

**Authors:** Amir Khorram-Manesh, Maxim A. Dulebenets, Krzysztof Goniewicz

**Affiliations:** 1 Department of Surgery, Institute of Clinical Sciences, Sahlgrenska Academy, Gothenburg University, 413 45 Gothenburg, Sweden; 2 Department of Development and Research, Armed Forces Center for Defense Medicine, Gothenburg, 426 76 Västra Frölunda, Sweden; 3 Department of Civil & Environmental Engineering, College of Engineering, Florida A&M University-Florida State University (FAMU-FSU), 2525 Pottsdamer Street, Building A, Suite A124, Tallahassee, FL 32310-6046, USA; mdulebenets@eng.famu.fsu.edu; 4 Department of Aviation Security, Military University of Aviation, 08521 Deblin, Poland; k.goniewicz@law.mil.pl

**Keywords:** contact tracing, ethics, pandemic, psychology, public education, public health

## Abstract

In the absence of a specific treatment or vaccines, public health strategies are the main measures to use in the initial stages of a pandemic to allow surveillance of infectious diseases. During the ongoing global pandemic of coronavirus disease 2019 (COVID-19), several countries initiated various public health strategies, such as contact tracing and quarantine. The present study aims to conduct a systematic literature review to identify the presence of educational initiatives that promote the implementation of public health strategies before public health emergencies, with a special focus on contact tracing applications. Using Science Direct, PubMed, Scopus, and Gothenburg University search engines, all published scientific articles were included, while conference, reports, and non-scientific papers were excluded. The outcomes of the reviewed studies indicate that the effective implementation of public health strategies depends on the peoples’ willingness to participate and collaborate with local authorities. Several factors may influence such willingness, of which ethical, psychological, and practical factors seem to be the most important and frequently discussed. Moreover, individual willingness and readiness of a community may also vary based on the acquired level of knowledge about the incident and its cause and available management options. Educational initiatives, proper communication, and timely information at the community level were found to be the necessary steps to counteract misinformation and to promote a successful implementation of public health strategies and attenuate the effects of a pandemic. The systematic review conducted as a part of this study would benefit the relevant stakeholders and policy makers and assist with effective designing and implementation.

## 1. Introduction

The unpredictable development of the coronavirus disease 2019 (COVID-19) resulted in a global pandemic beginning in 2020, spreading from one region to another [1]. Consequently, countries had to rely on traditional public health measures such as isolation, containment, quarantine, and contact tracing strategies [2,3,4,5,6]. However, these urgently implemented measures had varying success in halting the spread of the disease globally, either due to shortcomings in countries’ infrastructure or their financial capabilities. New waves of viral infection started spreading again as soon as society opened up [3,6].

In response to the worsening situation, several countries started various means of contact tracing and isolation of their citizens [7,8,9,10,11,12]. Contact tracing represents one strategy, which slows down the spread of the viral infection and enables the identification of infected cases or those at risk, either by listing all people with whom an infected person has recently been in contact, or by using location-tracking mobile applications (apps) [7,8,9,10,11,13,14,15,16]. There has been an intensive development of contact tracing applications (CTA), using various means of technology, including QR (Quick Response) codes; GPS (Global Positioning System); credit card transmission log; CCTV (Closed Circuit Television); different COVID alert apps; and Bluetooth [1,10,11,12,13,14,15,16,17,18,19,20]. However, the inadequate level of public compliance with these public health strategies has resulted in mandatory contact tracing strategies as part of a government strategy in some countries [8,17,21]. Within the European Union (EU), in some nations, authorities together with telecommunications providers started sharing people’s anonymous location data on map concentrations, respecting the Europe’s privacy laws and individuals’ rights to privacy [8,9,21,22,23,24,25,26,27].

Although the use of these apps seems necessary and beneficial, they seem to create several challenges, such as ethical, psychological, and practical, that may influence the willingness of individuals and a community to implement these measures actively [22,23,25]. The willingness itself is proven to be dependent on the level of information and knowledge, especially when dealing with unknown threats [28]. Adequate knowledge about incidents, their etiology, and the available management options enables recognition of the threats and acceptance of necessary strategies during public health emergencies. Consequently, community preparedness and mental readiness seem to be some of the most important elements in successful policy implementation [20,29,30,31,32,33,34,35,36,37,38,39,40,41,42,43,44]. It is therefore evident that investing in local empowerment by establishing educational initiatives, proper communication, and timely information are all necessary steps to counteract misinformation and to promote successful implementation of public health strategies, which will further attenuate the pandemic.

The present study aims to conduct a systematic literature review to identify the presence of educational initiatives that promote the implementation of public health strategies before public health emergencies, with a special focus on contact tracing applications. It also aims at opening discussions and creating a basis for the exchange of information from the countries implementing similar solutions, especially European countries, with which joint actions could be undertaken.

## 2. Materials and Methods

This review was conducted in accordance with the Preferred Reporting Items for Systematic Reviews and Meta-Analyses (PRISMA) guidelines and its flow diagram [45]. The searching process included articles and the PRISMA checklist for each considered study and abstract were completed and attached to this manuscript (Appendix A, Appendix B and Appendix C). The scientific evidence of each selected article was assessed, by using the Health Evidence Quality Assessment Tool (Appendix D), as Strong, Medium, and Weak [46]. Initially, Google Scholar was used as the testbed to estimate the number of hits and adjust the searching keywords accordingly. In the next step, Science Direct, Scopus, PubMed, and Gothenburg University’s Super search engines were used for a systematic search. The inclusions criteria were original research studies published in English. The exclusion criteria were conference papers, abstracts, reports, and non-scientific publications.

The research group performed the initial screening of all abstracts and titles independently to determine whether to include or exclude an article based on pre-defined selection criteria. The content analysis method was used to assess the eligibility of included papers, focusing on similarities and differences in the findings to present the tentative results [47]. During the abstract and title screening phase, a level of agreement on inclusion and exclusion was achieved among the authors. The third author reconciled disagreements (if any) between the first two authors to achieve a mutual consensus before moving to the full-text review. The full-text articles were assessed for inclusion, and the reasons were documented for all the excluded papers. The outcome was grouped based on the content analysis into four topics: practical, ethical, psychological, and educational aspects of public health emergency measures. Appendix A shows the combination of used search keywords and Table 1 illustrates the information about included studies. The key terms are as follows:

- Public Health Strategies
- Public Health Emergency
- Contact Tracing
- Isolation
- Quarantine
- Public Education

A standard data extraction form was used to collect the authors, article title, year published, journal title, study design, brief description of methods, primary outcome measures, and conclusions by all the authors for the articles included for full-text inclusions in the last step. References of the papers initially found were not included for evaluation. The search results from each database were exported to Microsoft Excel, merged, and sorted for removal of duplicate citations. Each article’s reference list was checked to identify reliable and relevant articles for inclusion into the final review list.

## 3. Results

**Figure 1:**
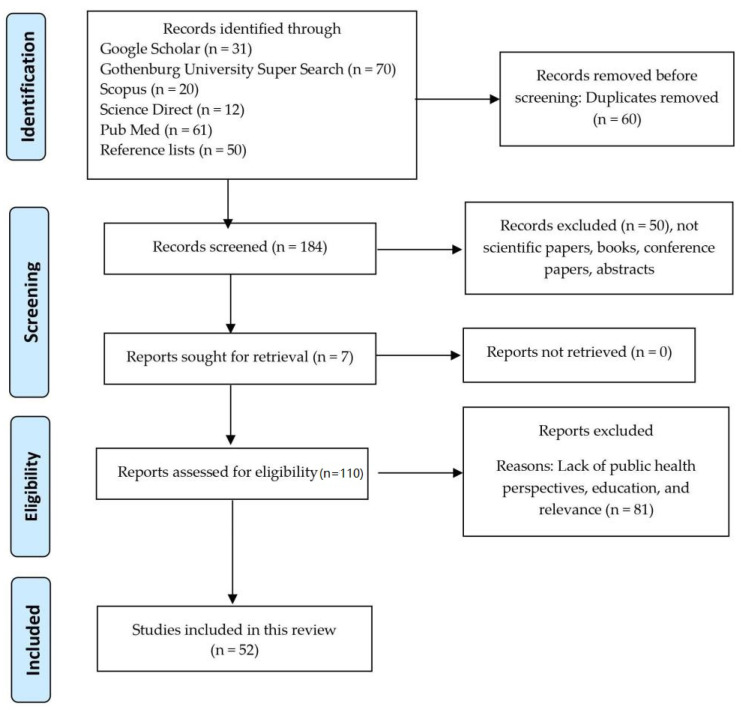
PRISMA 2020 flow diagram for new systematic reviews [44].

The initial term use, Public Health Strategies, returned over 2 million hits in Google Scholar. Similar results were also obtained using other search engines. The search term was changed to “Public Health Strategies” to receive a lower and manageable number of references (Appendix A). Other keywords were added stepwisely, and the number of hits decreased consequently. A descriptive summary of the results from the conducted systematic literature review is presented in Figure 1 in a PRISMA flow chart format. More details regarding the reviewed studies are presented in Table 1, including the following information: (1) health evidence (classified as either Strong (S) or Medium (M) or Weak (W)); (2) author(s); (3) year; (4) journal; and (5) subject of the study. The following four topics were revealed through the content analysis: (1) practical aspects; (2) ethical aspects; (3) psychological aspects; and (4) educational initiatives at the community level. The insights regarding these topics are discussed in the following sections of the manuscript.

### 3.1. Practical Aspects

A practical app should enable the identification of people who not only belong to the participants’ network but also those not known to them, such as fellow passengers on a bus. Working properly, a contact tracing app needs to comply with some technical requirements, of which the most important might be the need to operate at a close range. It is also important that many people use the technology to attain valid and accurate data. Some apps may prompt an individual who may have been in contact with a COVID-19 positive person to self-isolate [48].

Many different technological alternatives were discovered throughout the conducted systematic literature review, including GPS, Bluetooth, cellular location tracking, and QR codes, with a variety of practical and ethical concerns [49,50]. A GPS app, such as the one used in Korea, tracks the movement of people ordered to quarantine. However, the level of accuracy by which it traces contacts is around 10–20 m, which is not effective, particularly inside buildings. The precision of the cellular location data is even lower and poses significant concerns over the privacy of users. Some countries have used the QR codes to develop surveillance technologies. People are forced to scan their QR code doing certain activities (e.g., shopping, using public transportation) or when they enter into a new country. Although a QR-based system can efficiently control and restrict people’s movement, it can neither determine whether there has been any close contact between an infected individual and another person, nor if they obey and follow the rules of social distancing measures in public [49,50].

One promising solution, supported by the EU, is the use of Bluetooth to track the population. One important characteristic of Bluetooth is its ability to operate effectively at a close range. This, in turn, would facilitate the development of an application that allows users to opt-in or out easily by simply turning their Bluetooth on or off. Besides contact tracing, Bluetooth can also hypothetically help to control and measure whether the public respects the guidelines of social distancing. An important characteristic of Bluetooth, in contrast to other forms of cellular data, is its ability to function with an acceptable degree of accuracy at around 2 m. Consequently, its effective administration and utilization would potentially facilitate a faster return to normalcy [8,9,49,50].

Simple and safe technology can contribute to an increased willingness and ability of people to participate and thus increase the reliability and impact of the system. In Sweden, the number of people over 18 years having a smartphone is around 6.7 million. A total of 187,139 people had downloaded the recommended COVID-19 contact tracing app, introduced on April 29 by the end of July 2020 (2.8%) [17], while other countries have reported a higher number of participants (37%). However, these numbers are significantly lower than the recommended 65–70% to cover the necessary area and deliver a reliable result [50].

### 3.2. Ethical Aspects

Emerging evidence from some EU countries, most affected by the current pandemic, suggests that they use aggregated call detail records (CDRs) to carry out their stay-at-home policies and implementation of lockdowns [4,6,24,51]. In addition, there are other valid ethical concerns regarding the use of contact tracing apps within the EU, challenging its General Data Protection Regulation (GDPR).

According to the GDPR regulation, people have the possibility of using their rights in not disclosing whom they have been in contact with or can legally resist and challenge the tracking by authorities [7,19,22,35]. Furthermore, critics of contact tracing have pointed out that most of the apps are inconsistent with a range of older Android devices. Potentially, this inconsistency jeopardizes and influences the most vulnerable groups since elderly and poor citizens cannot use the new technology and lack the needed financial support to get one. There is also a lack of a mechanism to opt-in or opt-out of the third-party trackers in most used systems.

There has been a discussion regarding the pros and cons of a centralized vs. decentralized data collection system. In a centralized model, the process of matching occurs on a computer server, while in a decentralized model, the exchange takes place on people’s devices. Some claim that the relevant authorities will hold the data for a short period and handle it according to the highest ethical and security standards. However, there are reports arguing the necessity of undertaking a transparent Data Protection Impact Assessment (DPIA) before processing any personal data since data needs to be shared because of its nature. According to the EU Data Protection Watchdog, one of the requirements for using a Bluetooth app is to ensure its ‘privacy-by-design’. Finally, there is a need for a new legislation to facilitate and safeguard a return to normalcy once the crisis is over to guarantee the public trust enough to consider joining such mass surveillance strategies [7,8,9,51,52].

### 3.3. Psychological Aspects

The practical concerns about using apps may also get more complicated with the psychology behind using (or not using) any apps [53,54,55,56,57,58,59]. In a recent publication, Williams et al. [53] reported that the participants in their study were not sure whether using digital contact tracing was a good idea. Their moral reasoning and beliefs strongly influenced their standpoints. There were several themes in their reasoning, such as lack of information and misconceptions around the COVID-19 tracing app, concerns over privacy, stigma, and uptake, and contact tracing as a benefit for the population. These factors, particularly the concerns over privacy, stigma, and uptake, may create a psychological defense barrier for the willingness of using the app. Another Irish study confirmed the results from the UK, although the public willingness to download and use the app in that study was twice as high [53,59].

The use of new technologies and its impact on individuals may differ due to age or underlying medical and psychological conditions, and may include difficulties in the adoption of precautionary measures and altered daily routines. In one study, for instance, although the information and technology use kept the participants informed and connected, they experienced negative emotional consequences, including stress, worry, and anxiety, and reported varying degrees of preparedness [54]. In addition, rumors spread may influence public’s emotions, increasing their anxiety and anger [55]. Furthermore, isolation and quarantine during the pandemic seem to be associated with interpersonal sensitivity, somatization, and distress, especially symptoms like suspiciousness, hostility, and fearful thoughts of losing autonomy as well as feelings of inadequacy, uneasiness, and discomfort during interpersonal interactions [56]. All these issues result in poor adherence to self-isolation, contact tracing, and obeying recommendations [57].

### 3.4. Educational Initiatives at the Community Level

Although there are some variations in rate, over 75% of the populations within different EU countries have downloaded contact tracing apps. It is, however, not clear whether the intent to download corresponds to the actual download and use of the app [59]. Having in mind that information is a necessary part of the implementation process of new ideas and technology, particularly those of global interests, the need for educational initiatives to enable correct understanding of the use and benefits associated with the technologies is undeniable.

Previous studies concerning the 2009 H1N1 pandemic response have indicated that the primary barrier to successful management, besides overcrowding in houses, insufficient human resources, and the lack in local surveillance, is the inadequate community awareness regarding disease processes and prevention [60,61]. Other studies have also shown that adding community specific details, such as information about supplies and resources and details of how, when, where, and who has the responsibility for implementing recommendations outlined in the pandemic plans, together with the roles and responsibilities of the involved organizations, are essential elements in a pandemic plan and guarantee a successful outcome [62]. Irrespective of cultural and traditional background of each community, there have been successful efforts in low- and middle-income countries to engage and mobilize community members in case-detection and reduce the extent of infectious disease by establishing community-based strategies, including workshops and the use of social media [63]. One major facilitator for community engagement today is the use of new technology. Digital approaches, health technologies, and informatics might be used to inform all community members of the scale and development of pandemic, while they may also be designed and implemented to support public health surveillance and critical responses to adults’ and children’s well-being [64].

**Figure 2:**
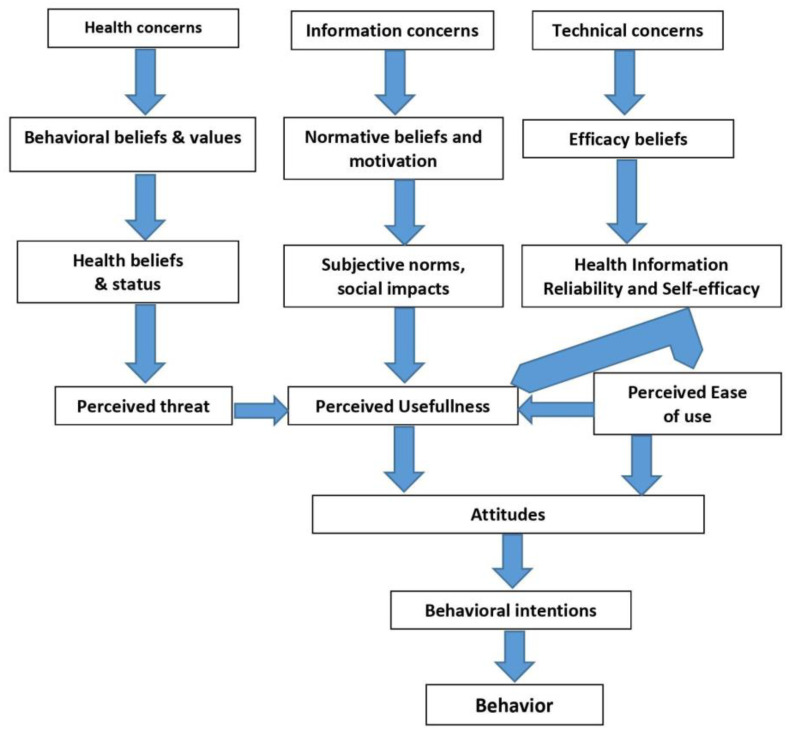
Health information technology acceptance model [65].

While a successful app should have certain grades of quality, security, privacy, defined usability, and compatibility, it is equally important that its need is matched to consumers’ general and health literacy levels. Kim and Park presented the concept of the health information technology acceptance model in 2012 (Figure 2) [65]. According to this model, each individual has three concern zones which influence individuals’ acceptance of technologies such as contact tracing apps. These are health concerns, information concerns, and technical concerns. All these concerns may influence an individual to accept or deny the use of an application. Within the health zone, the primary factor that influences the use of the app is how individuals perceive the usefulness of the app to their health. The result of the perceived threats creates a psychological incitement for using the new technology. Within the information zone, there are two primary factors: subjective norms and the technology’s credibility. The former is important for behavioral induction and consists of social pressure and community competition, i.e., the signals an individual receives from the surrounding networks about the society’s standing in accepting and using the new technology. Therefore, within the health technology, social networking is used to change consumers’ behavior and to predict their attitudes. Finally, factors, such as output quality, result demonstrability, objective usability, and perceived enjoyment, all demonstrating technological superiority, result in perceived ease of use and increased motivation for the use of an app [65].

## 4. Discussion

The efficiency of contact tracing in any form depends on the public’s willingness to participate and collaborate with the authorities. Such collaboration depends on the trust they have in the government regarding the safety and security measures they impose to protect their private lives and identity. Nowadays, societies with advanced and developed infrastructure are equally affected by the pandemic and might be the first victims [1,3,29,30,31]. One significant characteristic for these societies is their share in technical instruments and devices utilization. Mobile phones, Bluetooth, GPS, etc. not only enable social networking but also create ethical and psychological dilemmas that need to be discussed if using them as digital public health measures.

The current pandemic manifested at an accelerating rate and created a condition when the lack of specific treatment and vaccine necessitated the implementation of public health strategies. Such strategies may vary due to the cause of outbreak, but in a pandemic, may constitute social distancing, contact tracing, personal protective equipment, isolation, and quarantine [66]. Although the implementation of these strategies might be inevitable, they may create societal and human rights issues, which would yield to inadequate levels of compliance and different outcomes in various nations [12,40]. For instance, the social acceptance of implementing public health strategies has differed between Canada and the USA. The former is known as the home of social solidarity and the latter is known as a nation with rugged individualism, self-reliance, nonconformity, and independence [67]. Although social restrictions might be one of the main reasons for disobeying public health recommendations, other factors, such as housing, transportation, education, employment, food, and other household needs, are all crucial needs of persons under public health surveillance. Addressing these factors will increase individual willingness to comply with voluntary and mandated public health measures such as quarantine [68].

Technical requirements that include opt-in measures are necessary to grant contact tracing app users the ability to return to their normal lives in an achievable time, provided they use the app and respect other important public health measures. This would comply with the European Data Protection Board (EDPB)’s statement that restrictions of freedom during the pandemic or any other emergency during a strictly limited period are acceptable [31,32]. A solely Bluetooth-enabled app would operate on a system of individual anonymous codes, which enables the exchange of codes through a decentralized system. It gives users the most control over their data and should help them prevent the potential privacy conflicts that might arise if governments were to monitor the location data by teaming up with the relevant service providers such as Google. However, the Bluetooth technology still has its limitations.

The current COVID-19 pandemic and the development and deployment of digital public health technologies have initiated efforts to produce scientific and ethical sound guidelines and policies to guarantee personal information safety and ensure widespread public trust and uptake. Since ethical and legal aspects of using such technologies are also main concerns, one measure could be to conduct an ethical–legal analysis of these concepts with procedural considerations in technology governance. Such an approach may result in guidelines, navigation aids, or other algorithms that might help decision makers to ensure procedural validity and minimize ethical issues and shortcomings during the development or deployment of digital public health technologies [33].

Irrespective of all quality controls and information that may eliminate misconceptions, an improvement in psychology and an increase in the understanding of app utilization should be the first steps in using any apps, including contact tracing apps. Moreover, consumers should be educated regarding the content, benefits, and harms of these apps [69,70]. This approach would increase the perceived usefulness and ease of use but also indirectly increase the awareness of individuals in detecting new threats. Several reports have indicated that people with lower health literacy have worse healthcare and poorer health outcomes. They simply lack the skills necessary to manage their health and participate in disease prevention actively [71,72]. Previous studies in different parts of the world have shown that low or limited health literacy in the US, Southeast Asia, and the European Union are prevalent and consistently associated with several factors such as education, ethnicity, and age. In Europe, one in every two Europeans may not be able to comprehend essential health-related information and materials [72,73,74,75].

Key components necessary for the preparation of a community to combat emergencies are those of risk communication and community engagement strategies at the early stages. Preventive and emergency response strategies should be coordinated and strengthened at the community level by improving community resilience and through educational initiatives. The crucial steps in achieving a resilient and knowledgeable community are a transparent and trustful government–people relationship, improved health systems security proactivity, community to individual confinement, trust, and resilient solutions [76,77].

Furthermore, it is equally necessary that those working within the field of information privacy and security accommodate the public demands and protect their rights to privacy. A future public health emergency may not facilitate any option to adopt such mass surveillance measures [32]. It is, thus, crucial to ensure that policies, mathematical models, and technological measures are developed and in place to protect the collected and used data and promote transparency in how data can help contain the spread of disease while protecting and ensuring civil liberties [31].

## 5. Limitations

The main limitation of this study is its focus on the published literature in English. Consequently, relevant information in other languages may be missing. The criteria used to narrow the selection of included publications enabled the authors to access eligible data and a feasible number of publications to handle the content analysis and to perform the review. However, the criteria used may have been too selective, resulting in missing information. These limitations can be further addressed as a part of the future research.

## 6. Conclusions

A public health situation becomes an emergency when its scale, timing, or unpredictability have the potential to overwhelm routine capabilities. Such a definition necessitates an all-hazards approach to preparedness, allows for the optimal development of capabilities across scenarios, and better prepares communities for a broad spectrum of potential risks [1,78]. As public health emergencies, in general, and pandemics, in particular, are on the rise, the use of technologies in future communicable diseases might be inevitable. However, it is evident that there are some practical, psychological, and ethical challenges that need to be resolved before future public health policy planning [33]. The implementation of public health strategies, in the absence of appropriate treatment or vaccine, demands higher public health knowledge to recognize, accept, and deal with all restrictions and concerns. Besides educational initiatives at all levels of the society, particularly at the community levels, policy makers should also be prudent in evaluating the risk and benefits of using such technologies while technicians should determine how new generation technologies could be effectively adjusted to increase the public trust when using various technology-based public health strategies in increasing global disorder.

## Figures and Tables

**Table 1:** Shows the included studies, health evidence, information about authors and their affiliation, year of publication and the main topic of discussion, journal, and subject description. Topics are shown as E = Ethical, Ed = Educational, P = Practical, and Ps = Psychological.

No.	Health Evidence	Author(s)	Year	Topic	Country	Journal	Subject
1	M	Nelson et al.	2007	P	USA	*Am J Public Health*	Conceptualizing and defining public health emergency preparedness
2	M	Charania et al.	2011	Ed, P	Canada	*Int J Circumpolar Health*	The 2009 H1N1 pandemic response in remote First Nation communities of Subarctic Ontario: barriers and improvements from a health care services perspective
3	S	Charania et al.	2012	Ed, P	Canada	*BMC Public Health*	A community-based participatory approach and engagement process create culturally appropriate and community informed pandemic plans after the 2009 H1N1 influenza pandemic
4	S	Kim et al.	2012	Ed, Ps	S. Korea	*J Med Intern Res*	Development of a health information technology acceptance model using consumers’ health behavior intention
5	S	Cantey et al.	2013	Ed, P	USA	*J Public Health Manag Pract*	Public health emergency preparedness: lessons learned about monitoring of interventions from the National Association of County and City Health Official’s survey of nonpharmaceutical interventions for pandemic H1N1
6	M	Rothstein	2015	E, P	USA	*SSRN. Ind Health Law Rev*	Legal and ethical considerations for modern quarantine
7	M	Bachtiger et al.	2020	P, Ps	UK	*medRxiv*	Government policy and reduced willingness to participate in app-based contact tracing
8	S	Joo et al.	2020	Ed, P, Ps	S. Korea	*Service Business*	Resolving the tension between full utilization of contact tracing app services and user stress as an effort to control the COVID-19 pandemic
9	M	Khorram-Manesh et al.	2020	E, P, Ed	Sweden	*Disaster Med Public Health Prep*	Association between welfare, developed infrastructure and prosperity of a country with infectious disease spread
10	M	Alanezi et al.	2020	P, Ed	Saudi Arabia	*J Healthcare Leadership*	A comparative study on the strategies adopted by several countries to contain the spread of the COVID-19 pandemic
11	M	Nazareth et al.	2020	P, Ed	UK	*Lancet*	Early lessons from a second COVID-19 lockdown in Leicester, UK
12	M	Abeler et al.	2020	P, E	UK	*JMIR mHealth uHealth*	COVID-19 contact tracing and data protection
13	M	Hernandez-Quevedo et al.	2020	P, E	European Union	*The Health System Response Monitor*	How do countries structure contact tracing operations and what is the role of apps?
14	M	Du et al.	2020	P, E	China	*JMIR*	COVID-19 contact tracing apps and gaps for international pandemic control
15	S	Korea CDC	2020	P	S. Korea	*Osong Public Health Res*	Contact transmission of COVID-19 in South Korea and novel investigation techniques for tracing contacts
16	M	Rowe et al.	2020	E, P	France	*Eur J Info Sys*	Contact-tracing apps and alienation in the age of COVID-19
17	S	McGrail et al.	2020	E, P, Ps	Canada	*AJPH*	Contact-tracing apps and broader societal change
18	S	Bernard et al.	2020	E, Ps	UK	*AJPH*	An examination of the rise in government surveillance through mobile applications
19	M	Maghdid et al.	2020	P	Iraq	*SN Computer Science*	A smartphone-enabled approach to manage COVID-19 lockdown and economic crisis
20	M	Li et al.	2020	P	China	*Lancet*	Active case finding and case management: the key to tackling the COVID-19 pandemic
21	S	Guillon et al.	2020	E, Ps	France	*Public Health*	Attitudes and opinions on quarantine and support for a contact-tracing application in France during the COVID-19 outbreak
22	M	Hager et al.	2020	E, P	European Union	*Int Political Sociolog*	Collective discussion: toward critical approaches to intelligence as a social phenomenon
23	M	Abuhammad et al.	2020	E, Ed, P	Jordan	*Patient Preference and Adherence*	COVID-19 contact-tracing technology: acceptability and ethical issues of use
24	M	Reimer et al.	2020	E, P	Australia	*Eur J Info Sys*	Digital contact-tracing adoption in the COVID-19 pandemic
25	M	Basu et al.	2020	E	India	*Camb Quart Healthcare Ethics*	Mobile phones and contact tracing
26	W	Colins	2020	E, P	USA	*Mondaq Bus Brief*	Evolving considerations for multinational employers
27	W	Maati et al.	2020	E, P	Germany	*Czech J Int Relations*	Framing the pandemic and the rise of the digital surveillance state
28	M	Ekong et al.	2020	E, P	Nigeria	*JMIR Mhealth Uhealth*	Mobile positioning data contact tracing and patient privacy regulations
29	M	Dong et al.	2020	E, Ps	China	*J Med Internet Res*	Public emotions and rumors spread during the COVID-19 epidemic
30	S	Sfendla et al.	2020	Ps	Morocco	*Health Secur*	Factors associated with psychological distress and physical activity during the COVID-19 pandemic
31	S	Smith et al.	2020	E, P	UK	*Public Health*	Factors associated with adherence to self-isolation and lockdown measures in the UK: a cross-sectional survey
32	M	Shah et al.	2020	E	Nepal	*Nepal Med Assoc*	Combating the COVID-19 pandemic in Nepal: Ethical challenges in an outbreak
33	M	O’Callaghan et al.	2020	E, P	Ireland	*Ir J Med Sci*	A national survey of attitudes to COVID-19 digital contact tracing in the Republic of Ireland
34	M	Ye	2020	P, Ps	US	*JMIR Pediatr Parent*	Pediatric mental and behavioral health in the period of quarantine and social distancing with COVID-19
35	M	Rothstein	2020	E, P	US	*J Law Med and Ethic*	The coronavirus pandemic: public health and American values.
36	M	Torous et al.	2020	P, Ps	US	*JMIR Mental Health*	Digital mental health and COVID-19: using technology today to accelerate the curve on access and quality tomorrow
37	S	Tambo et al.	2020	Ed, P	Cameron	*Global Health J*	Early stage risk communication and community engagement (RCCE) strategies and measures against the coronavirus disease 2019 (COVID-19) pandemic crisis
38	W	Simon	2020	E	USA	*Survival*	Subtle connections: pandemic and the authoritarian impulse
39	M	EU	2021	E, P	European Union	*Commissioners’ Office Web*	Guide to the general data protection regulation (GDPR)
40	M	Shuja	2021	E	Pakistan	*Applied Intelligence*	COVID-19 open source data sets
41	M	Baumgart et al.	2021	Ed, P	Canada	*NPI Digital Medicine*	Digital advantage in the COVID-19 response: perspective from Canada
42	M	Jacob et al.	2021	P	Canada	*Policy design and practice*	The adoption of contact tracing applications for COVID-19 by the European governments
43	S	Sowmiya et al.	2021	E	India	*SN Computer Science*	A survey on security and privacy issues in contact tracing applications
44	M	Gerli et al.	2021	P	UK	*Government Info Quarterly*	The public value of eHealth application in a pandemic
45	M	Niccolai et al.	2021	P	USA	*AJPH*	Rapid establishment of a volunteer **contact tracing** program for COVID-19
46	M	Thoung et al.	2021	P	Vietnam	*BMJ Global Health*	Public safety and response to the COVID-19 epidemic in Vietnam
47	M	Hassandoust et al.	2021	Ed, P	New Zealand	*JAMIA*	Individuals’ privacy concerns and adoption of contact tracing mobile applications in a pandemic
48	S	Bradshaw et al.	2021	Ps	Australia	*Frontiers in Psychology*	The information safety assurance increases intentions to use COVID-19 contact tracing applications
49	M	Legendre et al.	2021	P	Switzerland	*arXiv*	Contact tracing technologies and cyber risks
50	S	CDC	2021	P	USA	*Web*	Contact tracing resources
51	S	Williams et al.	2021	E, Ps	UK	*Health Expectations*	Public attitudes towards COVID-19 contact tracing apps
52	S	Chen et al.	2021	P, Ps	USA	*Geriatr Nurs*	Reactions to COVID-19, information and technology use, and social connectedness among older adults with pre-frailty and frailty

## Data Availability

Datasets used and analyzed during the current study are presented as Appendix A, Appendix B, Appendix C and Appendix D.

## Appendix A. Searching Procedures

**Search Engine**	**Searching Keywords**	**Hits**
**Google Scholar** **Reviewed 31 Duplicates 10, Irrelevant 19, Included 12**	Public Health Strategies	>4 million
	“Public Health Strategies”	21,000
	“Public Health emergency” AND [(Contact Tracing) OR (Isolation) OR (Quarantine)]	31,100
	“Public Health emergency” AND “[(Contact Tracing) OR (Isolation) OR (Quarantine)]”	6690
	“Public Health emergency” AND “Contact Tracing” AND “Isolation” AND “Quarantine” AND Public Education	2850
	“Public Health emergency” AND “[(Contact Tracing) OR (Isolation) OR (Quarantine)]” AND “Public Education”	354
	“Public Health emergency” AND “[(Contact Tracing) OR (Isolation) OR (Quarantine)]” AND “Public Education” AND “Community resilience”	31
**Gothenburg University Super Search** **Reviewed 70 Duplicates 50, Irrelevant 15, Included 7**	“Public Health emergency” AND “Contact Tracing”	2281
	“Public Health emergency” AND “Contact Tracing” AND “Isolation”	1223
	“Public Health emergency” AND “Contact Tracing” AND “Isolation” AND “Quarantine”	803
	“Public Health emergency” AND “Contact Tracing” AND “Isolation” AND “Quarantine” AND Public Education	437
	“Public Health emergency” AND “Contact Tracing” AND “Isolation” AND “Quarantine” AND “Public Education”	70
**Science Direct** **Reviewed 15, Irrelevant 10, Included 5**	“Public Health emergency” AND [(Contact Tracing) OR (Isolation) OR (Quarantine)]	456
	“Public Health emergency” AND “[(Contact Tracing) OR (Isolation) OR (Quarantine)]”	313
	“Public Health emergency” AND “[(Contact Tracing) OR (Isolation) OR (Quarantine)]” AND Public Education	218
	“Public Health emergency” AND “[(Contact Tracing) OR (Isolation) OR (Quarantine)]” AND “Public Education”	113
	“Public Health emergency” AND “Contact Tracing” AND “Isolation” AND “Quarantine” AND Public Education	15
**Scopus** **Reviewed 20, Irrelevant 13, Included 8**	“Public Health emergency” AND “Contact Tracing”	286
	“Public Health emergency” AND “Contact Tracing” AND “Isolation”	117
	“Public Health emergency” AND “Contact Tracing” AND “Isolation” AND “Quarantine”	63
	“Public Health emergency” AND “Contact Tracing” AND “Isolation” AND “Quarantine” AND Public Education	20
	“Public Health emergency” AND “Contact Tracing” AND “Isolation” AND “Quarantine” AND “Public Education”	0
**PubMed** **Reviewed 61, Irrelevant 33, Included 15**	“Public Health emergency” AND [(Contact Tracing) OR (Isolation) OR (Quarantine)]	>2 million
	“Public Health emergency” AND “[(Contact Tracing) OR (Isolation) OR (Quarantine)]”	466
	“Public Health emergency” AND “[(Contact Tracing) OR (Isolation) OR (Quarantine)]” AND Public Education	62
	“Public Health emergency” AND “[(Contact Tracing) OR (Isolation) OR (Quarantine)]” AND “Public Education”	0

## Appendix B. PRISMA Checklist Abstract

**Section and Topic**	**Item Number**	**Checklist Item**	**Reported (Yes/No)**
**TITLE**			
Title	1	Identify the report as a systematic review.	YES
**BACKGROUND**			
Objectives	2	Provide an explicit statement of the main objective(s) or question(s) the review addresses.	YES
**METHODS**			
Eligibility criteria	3	Specify the inclusion and exclusion criteria for the review.	YES
Information sources	4	Specify the information sources (e.g., databases, registers) used to identify studies and the date when each was last searched.	YES
Risk of bias	5	Specify the methods used to assess risk of bias in the included studies.	No
Synthesis of results	6	Specify the methods used to present and synthesise results.	No
**RESULTS**			
Included studies	7	Give the total number of included studies and participants and summarise relevant characteristics of studies.	No
Synthesis of results	8	Present results for main outcomes, preferably indicating the number of included studies and participants for each. If meta-analysis was done, report the summary estimate and confidence/credible interval. If comparing groups, indicate the direction of the effect (i.e., which group is favoured).	YES
**DISCUSSION**			
Limitations of evidence	9	Provide a brief summary of the limitations of the evidence included in the review (e.g., study risk of bias, inconsistency and imprecision).	YES
Interpretation	10	Provide a general interpretation of the results and important implications.	No
**OTHER**			
Funding	11	Specify the primary source of funding for the review.	None
Registration	12	Provide the register name and registration number.	None
From: Page M.J., McKenzie J.E., Bossuyt P.M., Boutron I., Hoffmann T.C., Mulrow C.D., et al. The PRISMA 2020 statement: an updated guideline for reporting systematic reviews. BMJ 2021;372:n71. doi:10.1136/bmj.n71.			

## Appendix C. PRISMA Checklist Manuscript

**Section and Topic**	**Item Number**	**Checklist Item**	**Location Where Item Is Reported**
**TITLE**			
Title	1	**Implementing contact tracing and other public health strategies; needs for educational initiatives—A Systematic Review**	Page 1
**ABSTRACT**			
Abstract	2	See the PRISMA 2020 for Abstracts checklist.	Page 1
**INTRODUCTION**			
Rationale	3	Describe the rationale for the review in the context of existing knowledge.	Page 1–3
Objectives	4	Provide an explicit statement of the objective(s) or question(s) the review addresses.	Page 1 & 3
**METHODS**			
Eligibility criteria	5	Specify the inclusion and exclusion criteria for the review and how studies were grouped for the syntheses.	Method page 2–3
Information sources	6	Specify all databases, registers, websites, organisations, reference lists and other sources searched or consulted to identify studies. Specify the date when each source was last searched or consulted.	Method page 2
Search strategy	7	Present the full search strategies for all databases, registers and websites, including any filters and limits used.	Appendix.
Selection process	8	Specify the methods used to decide whether a study met the inclusion criteria of the review, including how many reviewers screened each record and each report retrieved, whether they worked independently, and if applicable, details of automation tools used in the process.	Method page 2–3
Data collection process	9	Specify the methods used to collect data from reports, including how many reviewers collected data from each report, whether they worked independently, any processes for obtaining or confirming data from study investigators, and if applicable, details of automation tools used in the process.	Method page 2–3
Data items	10a	List and define all outcomes for which data were sought. Specify whether all results that were compatible with each outcome domain in each study were sought (e.g., for all measures, time points, analyses), and if not, the methods used to decide which results to collect.	Figure 1 page 4, Table 1, page 5
	10b	List and define all other variables for which data were sought (e.g., participant and intervention characteristics, funding sources). Describe any assumptions made about any missing or unclear information.	See point 10a
Study risk of bias assessment	11	Specify the methods used to assess risk of bias in the included studies, including details of the tool(s) used, how many reviewers assessed each study and whether they worked independently, and if applicable, details of automation tools used in the process.	Method page 2–3
Effect measures	12	Specify for each outcome the effect measure(s) (e.g., risk ratio, mean difference) used in the synthesis or presentation of results.	NA
Synthesis methods	13a	Describe the processes used to decide which studies were eligible for each synthesis (e.g., tabulating the study intervention characteristics and comparing against the planned groups for each synthesis (item #5)).	Result page 3–4
	13b	Describe any methods required to prepare the data for presentation or synthesis, such as handling of missing summary statistics, or data conversions.	NA
	13c	Describe any methods used to tabulate or visually display results of individual studies and syntheses.	-
	13d	Describe any methods used to synthesize results and provide a rationale for the choice(s). If meta-analysis was performed, describe the model(s), method(s) to identify the presence and extent of statistical heterogeneity, and software package(s) used.	Method page 2–3
	13e	Describe any methods used to explore possible causes of heterogeneity among study results (e.g., subgroup analysis, meta-regression).	-
	13f	Describe any sensitivity analyses conducted to assess robustness of the synthesized results.	-
Reporting bias assessment	14	Describe any methods used to assess risk of bias due to missing results in a synthesis (arising from reporting biases).	-
Certainty assessment	15	Describe any methods used to assess certainty (or confidence) in the body of evidence for an outcome.	Method page 2
			HEQAT
**RESULTS**			
Study selection	16a	Describe the results of the search and selection process, from the number of records identified in the search to the number of studies included in the review, ideally using a flow diagram.	Results, Page 3
	16b	Cite studies that might appear to meet the inclusion criteria, but which were excluded, and explain why they were excluded.	Figure 1
Study characteristics	17	Cite each included study and present its characteristics.	Page 5–7
Risk of bias in studies	18	Present assessments of risk of bias for each included study.	–
Results of individual studies	19	For all outcomes, present, for each study: (a) summary statistics for each group (where appropriate) and (b) an effect estimate and its precision (e.g., confidence/credible interval), ideally using structured tables or plots.	–
Results of syntheses	20a	For each synthesis, briefly summarise the characteristics and risk of bias among contributing studies.	–
	20b	Present results of all statistical syntheses conducted. If meta-analysis was done, present for each the summary estimate and its precision (e.g., confidence/credible interval) and measures of statistical heterogeneity. If comparing groups, describe the direction of the effect.	–
	20c	Present results of all investigations of possible causes of heterogeneity among study results.	–
	20d	Present results of all sensitivity analyses conducted to assess the robustness of the synthesized results.	–
Reporting biases	21	Present assessments of risk of bias due to missing results (arising from reporting biases) for each synthesis assessed.	–
Certainty of evidence	22	Present assessments of certainty (or confidence) in the body of evidence for each outcome assessed.	Table 1, page 5–7
**DISCUSSION**			
Discussion	23a	Provide a general interpretation of the results in the context of other evidence.	Page 9–11
	23b	Discuss any limitations of the evidence included in the review.	Page 9–11
	23c	Discuss any limitations of the review processes used.	Page 9–11
	23d	Discuss implications of the results for practice, policy, and future research.	Page 9–11
**OTHER INFORMATION**			
Registration and protocol	24a	Provide registration information for the review, including register name and registration number, or state that the review was not registered.	Page 11
	24b	Indicate where the review protocol can be accessed, or state that a protocol was not prepared.	Journals homepage
	24c	Describe and explain any amendments to information provided at registration or in the protocol.	–
Support	25	Describe sources of financial or non-financial support for the review, and the role of the funders or sponsors in the review.	None
Competing interests	26	Declare any competing interests of review authors.	None
Availability of data, code and other materials	27	Report which of the following are publicly available and where they can be found: template data collection forms; data extracted from included studies; data used for all analyses; analytic code; any other materials used in the review.	Journals homepage
From: Page M.J., McKenzie J.E., Bossuyt P.M., Boutron I., Hoffmann T.C., Mulrow C.D., et al. The PRISMA 2020 statement: an updated guideline for reporting systematic reviews. BMJ 2021;372:n71. doi:10.1136/bmj.n71. For more information, visit: http://www.prisma-statement.org/ (accessed on 25 April 2021).			
